# Baseline characterization of entomological drivers of malaria transmission in Namibia: a targeted operational entomological surveillance strategy

**DOI:** 10.1186/s13071-023-05822-0

**Published:** 2023-07-05

**Authors:** Ophilia Lukubwe, Tabeth Mwema, Rosalia Joseph, Deodatus Maliti, Iitula Iitula, Stark Katokele, Petrina Uusiku, Dennis Walusimbi, Sheila B. Ogoma, Cara Smith Gueye, Elodie Vajda, Allison Tatarsky, Edward Thomsen, Munya Tambo, Davis Mumbengegwi, Neil F. Lobo

**Affiliations:** 1 University of Science and Technology, Health and Applied Sciences, Windhoek, Namibia; 2 Multidisciplinary Research Center, University of Namibia, Windhoek, Namibia; 3 National Vector Borne Disease Control Program, Ministry of Health and Social Services, Windhoek, Namibia; 4 Clinton Health Access Initiative, Boston, Massachusetts USA; 5 Malaria Elimination Initiative, UCSF Institute for Global Health Sciences, University of California, San Francisco, San Francisco, California USA; 6 Eck Institute for Global Health, University of Notre Dame, Notre Dame, Indiana USA

**Keywords:** Entomological drivers, Malaria, Namibia

## Abstract

**Background:**

Namibia’s focus on the elimination of malaria requires an evidence-based strategy directed at understanding and targeting the entomological drivers of malaria transmission. In 2018 and 2019, the Namibia National Vector-borne Diseases Control Program (NVDCP) implemented baseline entomological surveillance based on a question-based approach outlined in the Entomological Surveillance Planning Tool (ESPT). In the present study, we report on the findings of the ESPT-based NVDCP on baseline vector species composition and bionomic traits in malaria endemic regions in northern Namibia, which has the aim of generating an evidence base for programmatic decision-making.

**Methods:**

Nine representative sentinel sites were included in the 2018 entomological surveillance program (Kunene, Omusati, Oshana, Ohangwena, Oshikoto, Otjozondjupa, Kavango West, Kavango East and Zambezi); the number was reduced to four sites in 2019 due to limited funding (Ohangwena, Kavango West, Kavango East, and Zambezi). In the 2018 baseline collections, multiple sampling methods (human landing catches, pyrethroid spray catches, U.S. Centers for Disease Control and Prevention light traps [CDC-LTs], resting boxes [RBs] and larval sampling) were utilized to evaluate indoor/outdoor human biting rates, resting behaviors and insecticide resistance (IR). CDC-LTs and RBs were not used in 2019 due to low and non-representative sampling efficacies.

**Results:**

Overall, molecular evidence demonstrated the presence of three primary mosquito vectors, namely *Anopheles arabiensis*, rediscovered *Anopheles gambiae* sensu stricto and *Anopheles funestus* sensu stricto, alongside *Anopheles squamosus* and members of the *Anopheles coustani* complex. Vectors were found to bite throughout the night (1800 hours 0600 hours) both indoors and outdoors, with *An. arabiensis* having the highest biting rates outdoors. Low numbers of indoor resting *Anopheles* point to possible low indoor residual spraying (IRS) efficacy—with *An. arabiensis* found to be the major vector species resting indoors. The IR tests demonstrated varying country-wide resistance levels to the insecticide deltamethrin, with the resistance levels confirmed to have increased in 2019, evidence that impacts national programmatic decision-making. Vectors demonstrated susceptibility to the insecticides dichlorodiphenyltrichloroethane, bendiocarb and Actellic 300CS in 2018, with mosquitoes from only one site (Kavango West) demonstrating possible resistance to DDT. Targeted and question-based entomological surveillance enabled a rapid and focused evidence base to be built, showing where and when humans were being bitten and providing entomological data on long-lasting insecticidal nets, IRS efficacy and insecticide resistance, which the Ministry of Health and Social Services-Namibia can use to further build a monitoring and evaluation framework for understanding the drivers of transmission.

**Conclusion:**

Identification and characterization of species-specific bionomic traits allows for an understanding of where and when vector human contact may occur as well as the potential impact of interventions. Low indoor resting rates as well as the presence of insecticide resistance (and the increase in its frequency) point to the need for mosquito-behavior-directed and appropriate interventions as well as the requirement for a resistance mitigation strategy. The ESPT-based question- and minimal essential indicator-based operational research strategy provides programs with directed and focused data for facilitating decision-making while requiring limited funding and capacity.

**Graphical Abstract:**

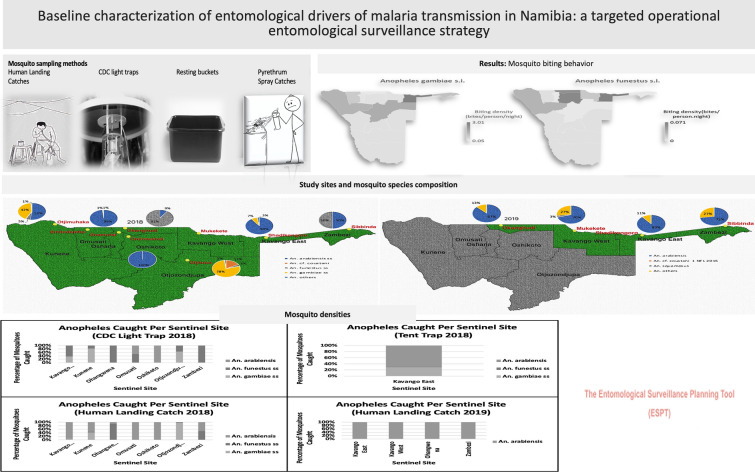

## Background

Over the last two decades, Namibia has seen a significant decline in malaria burden that has been attributed to the large-scale deployment of indoor residual spraying (IRS), the distribution and use of long-lasting insecticide-treated bed nets (LLINs), increased use of rapid diagnostic tests and treatment with artemisinin-based combination therapy [1, 2]. Malaria morbidity and mortality decreased by 98% over this period, far surpassing targets and enabling the implementation of elimination strategies. In 2009, the Elimination Eight (E8) initiative was launched, under which eight countries in southern Africa decided to collaborate to eliminate malaria in Namibia, Botswana, South Africa and Swaziland. Within the framework of this initiative, Namibia formally declared the ambition to eliminate malaria by 2020 [3]. However, success against malaria outbreaks in Namibia has plateaued, with cyclic outbreaks over the past 10 years. Malaria transmission is known to be clustered in the northern regions of the country [4], but evidence on the specific drivers of persistent transmission and how to address these gaps in protection would help guide elimination efforts.

In Namibia, malaria transmission occurs mainly in the endemic northern regions, contributing to almost the entire malaria burden in the country [5]. Malaria transmission occurs in nine of the 14 regions (22 districts) of Nambia, with 1.7 million people (approximately 68% of the population) at risk of contracting malaria. Namibia is stratified into three malaria transmission zones: zone 1, moderate transmission; zone 2, low transmission; zone 3, no risk [6]. Malaria transmission in Namibia is highly seasonal and varies from region to region, with an overall high transmission period occurring between January and May, after the rainy season [7]. Seasonal transmission occurs in the central north and north-western regions of the country, while endemicity is highest in the north-eastern regions of the country (Kavango East, Kavango West and Zambezi), characterized by year-round transmission coupled with seasonal peaks. As a result of this seasonality, malaria confers little or no immunity to people who are residing in the malaria-endemic areas [8].

In Namibia, the human protozoan parasite *Plasmodium falciparum* accounts for 97% of malaria cases, with the remaining 3% due to *Plasmodium vivax* [9]. Historical entomological baseline data collected in 1965 demonstrate the presence of *Anopheles gambiae* sensu lato (*An. gambiae* s.l.), *Anopheles funestus* and *Anopheles arabiensis* in Namibia [10]. However, more recent studies have shown a drastic reduction in endophilic *An. gambiae* sensu stricto (*An. gambiae* s.s.) and *An. funestus* sensu stricto (*An. funestus* s.s.) densities, which may be attributed to effective intra-domiciliary vector control, leaving exophilic *An. arabiensis* as the principal malaria vector [6, 11]. Thus, there remains a need to better characterize present-day vector presence and bionomic traits.

Vector control remains the key cornerstone of malaria control and elimination. In many parts of the world, the main vector control strategies used to control mosquito vector populations are targeted LLINs and IRS [12, 13]. Studies have shown IRS to be an effective vector control intervention in preventing and reducing malaria morbidity and mortality [14–16]. IRS is the main vector control intervention used to control malaria vectors and transmission in Namibia, with targeted LLIN distribution and supplementary larval source management (LSM) where applicable.

Despite annual programmatic records on vector bionomics, limited data are available on the composition, distribution and insecticide susceptibility of malaria vector species in Namibia. *Anopheles* mosquitoes were first reported in the 1950s by De Meillon, who reported that members of the *An. gambiae* s.l. and *An. funestus* sensu lato (*An. funestus* s.l.) complexes (based on morphological identification) were widely distributed in the country, especially in the northern regions [17]. In a study conducted in the early 1980s, Coetzee [18] discovered a new species, which he named *An. namibiensis*. Similarly, Ntomwa et al. [19] also identified members of the *An. gambiae* s.l. complex (i.e. *An. arabiensis*) and *An. funestus* s.l. complex (i.e. *An. funestus* s.s.). This latter study found that, when evaluating insecticide resistance (IR), *An. arabiensis* was ≥ 98% susceptible to the insecticides dichlorodiphenyltrichloroethane (DDT) and deltamethrin [9, 11]. To date, country-wide studies on the composition and distribution of the malaria vectors in Namibia have not been conducted. The results of such studies will be crucial to establishing the current state of malaria vector occurrence, as well as other important entomological indicators relevant for vector control operations.

No documented evidence on the IR of malaria vectors in Namibia has been obtained since 2018 [20]. However, the country has been implementing IRS for more than 16 decades, and it is therefore possible that IR has developed, possibly negatively impacting malaria vector control and elimination efforts. The development of IR poses a great threat to vector control programs [21], making annual insecticide susceptibility studies important for ensuring that vector control tools continue to be efficacious.

The Entomological Surveillance Planning Tool (ESPT) [22] is a decision-support tool for planning question-based entomological surveillance activities designed for the collection of minimum essential indicators to support cost-effective, locally tailored and evidence-based vector control. The ESPT enables malaria programs to quantify gaps in protection, such as spaces and times where and when individuals are exposed to vector bites, while also enabling in-country capacity building. In this study, we report on the findings from the ESPT-based National Vector-borne Diseases Control Program (NVDCP) on baseline vector compositions and bionomic traits in malaria endemic regions in northern Namibia, which has the aim to generate an evidence base for programmatic decision-making. This is the first demonstration of a standardized, NVDCP-led, ESPT-based entomological surveillance program in Namibia.

## Methods

### Applying the ESPT

The ESPT was piloted by the NVDCP in 2018 and 2019. The ESPT-based entomological surveillance plan was based on NVDCP’s priority program question: *‘What are the Anopheles species temporal compositions, bionomic characteristics, and susceptibility to insecticides in Namibia?’* The ESPT was used to select question-based minimal essential indicators and outline a sampling design grounded in available capacity, and served as a framework for data analysis and the interpretation of findings. Towards answering this question, several sampling methods were selected and evaluated in 2018 with the aim to capture key data elements, including species occurrence and density, human biting rate (HBR), biting time and location, indoor resting density and resistance frequency. Data-based evaluations of sampling methods in 2018 determined sampling methods utilized in 2019.

### Study sites

**Fig. 1:**
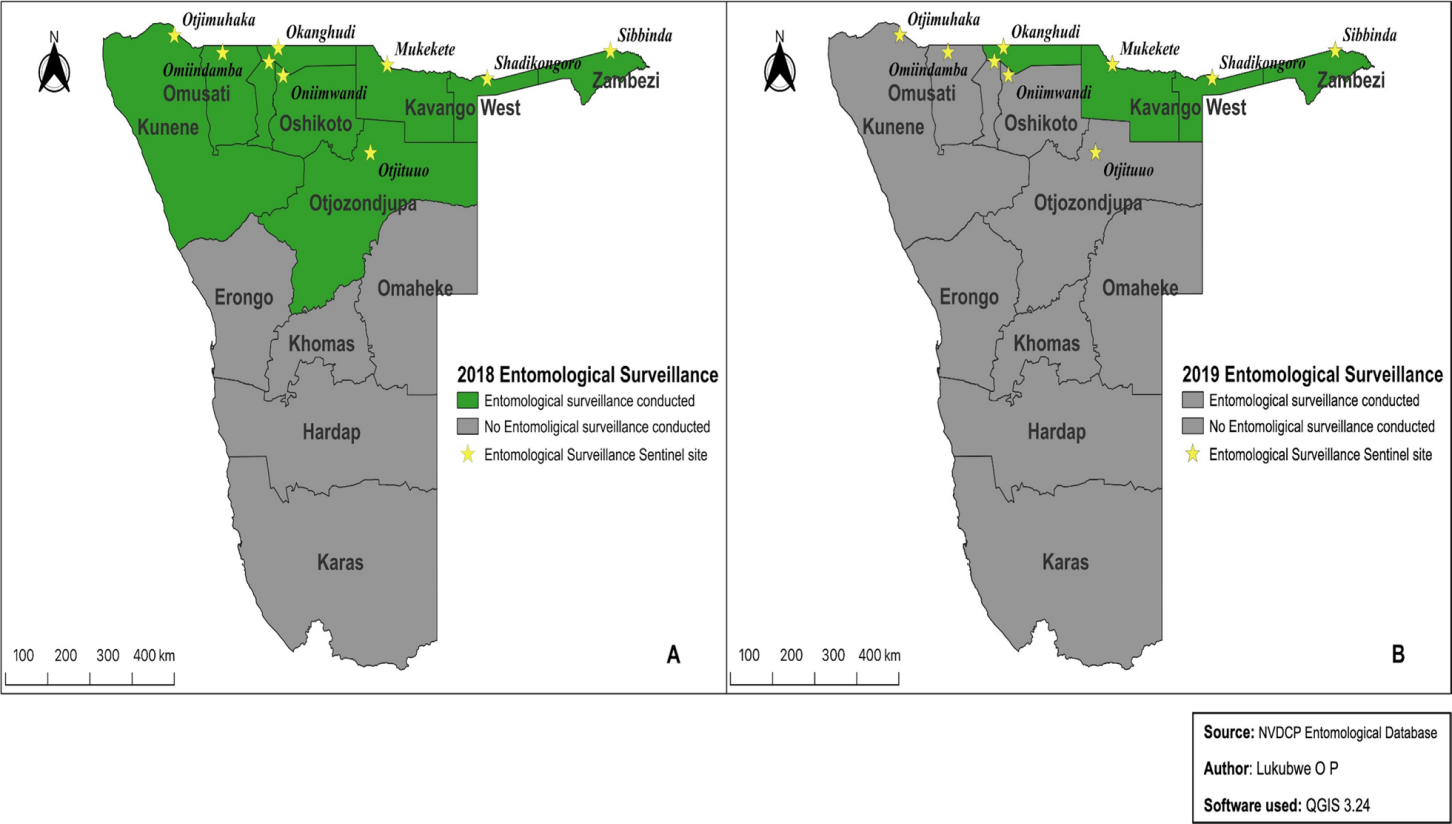
Maps of sentinel sites included in the 2018 (**a**) and 2019 (**b**) entomological surveillances. NVDCP, National Vector-borne Diseases Control Program

Annual entomological surveillance was conducted in 2018 and 2019 in nine and four sentinel sites, respectively, located in the malaria endemic regions of northern Namibia. The specific sentinel sampling site in each region was a malaria hotspot, as determined by the NVDCP based on malaria risk maps generated from malaria surveillance data of the past three consecutive years. Each sentinel site constituted a village in a malaria endemic region and included a sampling area of about 10-km radius from a central location of the village. The nine regions with the sentinel sites (in brackets) that were included in the 2018 entomological surveillance were Kunene (Otjimuhaka), Omusati (Etaka), Oshana (Onamutai), Ohangwena (Okanghudi), Oshikoto (Onayena), Otjozondjupa (Otjituuo), Kavango West (Mukekete), Kavango East (Shadikongoro) and Zambezi (Makanga) (Fig. 1a). The four sentinel sites studied in 2019 were Ohangwena (Okanghudi), Kavango West (Mukekete), Kavango East (Shadikongoro) and Zambezi (Sibbinda) (Fig. 1b).

### Sampling methods

Mosquito sampling was conducted between March and April (malaria transmission months) to determine the following entomological indicators: site-specific *Anopheles* species occurrence and density, indoor and outdoor HBRs, time of biting, indoor resting behavior and susceptibility to insecticides (deltamethrin, Actellic, bendiocarb, DDT) used in IRS. In 2018, a baseline survey was conducted in nine sentinel sites that represented a sampling village in each of the nine malaria endemic regions. In 2019, the same entomological parameters were studied between April and May but in only four out of the nine sentinel sites. The 2019 entomological surveillance was carried out in two rounds, with the first round conducted in April and the second round conducted in May.

#### Human landing catches

Human landing catches (HLCs) [23, 24] were conducted both inside and outside structures. A set of four sentinel households, with at least one type of each local house construction type represented (i.e. mud, zinc, traditional), was randomly selected from each sentinel village. Host-seeking adult mosquitoes were sampled for an entire night (from 1800 hours to 0600 hours) in each household, with variable numbers of nights based on the site and year ( as described below). Two community volunteers (CVs) sampled mosquitoes (one indoors and one outdoors) at each of the sampling households. The CVs collected landing mosquitoes for 45 min every hour, with resting breaks of 15 min in between collections, and swapped positions (inside and outside) every hour to minimize personal bias. A team of eight CVs worked a 6-h shift (1900 hours to 0100 hours) and were then replaced by another team of eight CVs for the remaining 6 h (a total of 16 volunteers were used per collection night). Mosquito samples from each household were placed in different holding cups and the cups labeled according to hour collected and location.

In 2018, there were a total of 16 trapping nights per site in Kavango East, Kavango West, Kunene, Ohangwena, Omusati, Oshana, Otjozondjupa and Zambezi and 12 trapping nights at the site in Oshikoto. There were two rounds of collections in 2019: in round 1, there were a total of 16 trapping nights per site each in Kavango East, Ohangwena and Zambezi in round 1 (March; summer) and only 12 trapping nights in Kavango West; in round 2, there were 16 trapping nights per site in all sites (May; autumn). Hence, there was a total of 32 trapping nights each in 2019 for Kavango East, Ohangwena and Zambezi and 28 trapping nights in Kavango West. In 2019 samples were collected in two sampling rounds to account for seasonal variation.

#### Pyrethrum spray catches

Houses where pyrethrum spray catches (PSCs) were conducted were not the same houses as those where HLCs were conducted; there was a buffer distance of at least 30 m between any house included in the entomology collections. Collections using PSCs were conducted in 2019 only. After first obtaining consent by the head of the household, field technicians removed all major obstacles in the rooms under study. All wall crevices and roof openings were covered with cloth to prevent mosquitoes from escaping. The floors of the rooms were then covered with white sheets and an insecticide (aerosol pyrethroid) was sprayed on the walls and roofs of the rooms for about 2 min. After 10 min, the white sheets were examined for knocked-down mosquitoes, which were collected and placed in holding cups. Different houses were sampled each night. There were two rounds of collections in 2019: in round 1, PSCs were conducted in a total of 16 houses each in Kavango East, Ohangwena and Zambezi, and only four PSCs were conducted in Kavango West; in round 2, there was a total of 16 PSCs in all sites. Hence, there was a total of 32 trapping nights each in 2019 for Kavango East, Ohangwena and Zambezi and 20 trapping nights in Kavango West.

#### U.S. Centers for Disease Control and Prevention miniature light traps

Sampling of host-seeking mosquitoes using U.S. Centers for Disease Control and Prevention miniature light traps (CDC-LTs) [25, 26] was conducted in two houses (houses in which other entomological sampling methods were not performed). For indoor sampling, a CDC-LT was hung 1 m above the floor and next to a bed in which a household member slept under a bed net. For outdoor sampling, a CDC-LT was hung about 10 m from a house where indoor sampling was conducted. CDC-LT catches were conducted from 1900 hour to 0700 hour. On the following morning, all mosquitoes trapped were removed from the trap and placed in holding cups. In 2018, there were a total of 16 trapping nights indoors and outdoors each in Kavango East, Kavango West, Kunene, Ohangwena, Omusati, Oshana, Oshikoto, Otjizondjupa and Zambezi. CDC-LT catches were not conducted in 2019.

#### Resting Box Collections

Two sentinel houses were used for catches with resting box collections (RBCs) [27, 28]. One resting container (a bucket lined with black cloth) was placed indoors and another was placed about 10 m away from the house. The RBs remained in their positions for 12 h (1900 hour to 0700 hour) after which mosquitoes found to rest inside the buckets were aspirated into holding cups. The total number of RB collection nights were site specific, with 16 indoor and 16 outdoor RB collections each in Kavango East, Kavango West, Kunene, Ohangwena, Omusati and Otjizondjupa, 12 indoor and 12 outdoor RB collections each in Zambezi and Oshikoto and only four indoor and four outdoor RB collections in Oshana. RB collections were not conducted in 2019.

#### Larval sampling and rearing

Larval sampling was conducted after the rainy season, during the months of March and April, using larval dippers [29–31]. Sampling of larvae was conducted in the same sentinel villages where adult mosquitoes were sampled. *Anopheles* mosquito larvae and pupae were identified morphologically and reared to adulthood at the NDVCP insectary in Oshakati and Zambezi. The larvae were fed on dog biscuits and yeast mixture, and emerging adults were fed on a 10% sugar solution ad libitum. Temperature and relative humidity within the insectary were maintained between 28 °C and 29 °C and 70–80%, respectively.

### Insecticide susceptibility assays

Larvae sampling for the insecticide resistance (IR) tests was conducted after the rainy season, during the months of March and April, using larval dippers [29–31]. Sampling of larvae was conducted in the same sentinel villages where adult mosquitoes were sampled. *Anopheles* mosquito larvae were identified morphologically and reared to adulthood at the NDVCP insectary in Oshakati and Zambezi under the same conditions as described in section Larval sampling and rearing. The WHO bioassays were conducted following WHO protocols [31]. Female *Anopheles* mosquitoes aged 3–5 days which had been raised from larvae and had never had a blood meal were used for the WHO IR bioassays. Susceptible *An. arabiensis* mosquitoes (KGB strain) were used for the control WHO bioassays. Live and dead mosquitoes at the end of the assay were separated and placed in tubes with labels specifying outcome. IR tests were conducted in 2018 across the nine sentinel sites for the following IRS insecticides deltamethrin 0.05%, DDT 4%, bendiocarb 1.25% and pirimiphos-methyl (Actellic) 0.25%. Resistance tests were done only in Zambezi in 2019 using deltamethrin 0.05%, DDT 4%, bendiocarb 1.25% and pirimiphos-methyl (Actellic) 0.25%.

### Species identification

All mosquitoes sampled were morphologically identified to species [32] where possible and placed individually in Eppendorf tubes containing silica gel separated from the mosquito by plain paper. PCR-based species identification was also conducted for morphologically identified members of the *An. gambiae* s.l. and *An. funestus* s.l. complexes [33, 34], while sequencing-based species identification using internal transcribed spacer 2 (ITS2) and cytochrome* c* oxidase subunit 1 (CO1) sequences [35] was conducted on a subsample of all specimens. Successful PCR identifications were obtained for only a portion of the collected mosquitoes (*n* = 1873 out of *n* = 3188). Therefore, we multiplied the number of *An. gambiae* s.l. without a confirmed molecular identification (disaggregated by village, biting location and hour) by the proportions of *An. arabiensis* and *An. gambiae* s.s. that were successfully identified at that location/time. Subsequent analyses used this estimated dataset.

### Analysis

Insecticide susceptibility data were analyzed according to WHO protocols. Variability in the number of anophelines caught by HLC was investigated using generalized linear mixed models (GLMM) with a negative binomial distribution and log link function. All analyses were performed in R v4.3.2 using the lme4 package (R Foundation for Statistical Computing, Vienna, Austria). Separate models were fit for each of the following response variables: total anophelines, *An. gambiae* s.s.*, An. arabiensis* and *An. funestus* s.s. Random effects included village, date and their interaction. For the total anopheline, *An. arabiensis*, and *An. funestus* s.s. models, fixed effects included biting location (indoors/outdoors), biting time, year and year × location interaction. For the *An. gambiae* s.s. model, fixed effects including the year variable were removed because *An. gambiae* s.s. was only caught in 2018. For the remaining descriptive statistics, mosquito catches were standardized to bites per night (bpn).

## Results

The ESPT was utilized to design and implement baseline entomological surveillance in malaria endemic regions of Namibia in 2018, with evidence-based adaptations in year 2 (2019). Key indicators on drivers of transmission collected include species occurrence and density, HBR, human biting activity (time and location), indoor resting density and resistance frequency. Sampling devices were also evaluated as part of the operational research.

### Species occurrence, density and HBR

Human landing catches provided the most data towards understanding species-specific occurrence, density and HBR. HLC samples included *An. gambiae* s.l., *An. funestus* s.l. and species not identified as part of the *An. gambiae* s.l. or *An. funestus* s.l. complexes (*Anopheles* ‘other’). Molecular identification using PCR diagnostics identified *An. arabiensis* and *An. gambiae* s.s. in the *An. gambiae* s.l. complex, and *An. funestus * s.s. in the *An. funestus* s.l. complex. Sequencing of a subsample of *Anopheles* ‘other’ samples using ITS2 and CO1 sequences identified *Anopheles squamosus* and members of the *Anopheles coustani* complex, namely *An. coustani* and *An. cf. coustani 1 NFL-2015* (Genbank Accession No. KR014841). The most abundant mosquitoes at all sentinal sites were *An. arabiensis*, *An. gambiae* s.s. and *An. funestus* s.s.. Site and location specific HBRs are reported in Table 1. *Anopheles squamosus* and *An. coustani* sensu lato specimens were grouped into *Anopheles* ‘other’ when determining HBRs. Each site demonstrated site-specific species and HBRs.

**Table 1:** Human landing catch* Anopheles* species composition and human biting rates standardized to bites per night by site

Region	Species	HBR (bpn) 2018			HBR (bpn) 2019 round 1			HBR (bpn) 2019 round 2		
		Indoor	Outdoor	Total	Indoor	Outdoor	Total	Indoor	Outdoor	Total
Kavango East	*An. arabiensis*	19.5	56.9	1222.0	2.3	12.8	241.0	10.0	28.1	610.0
	*An. gambiae* s.s	0.1	10.3	166.0	0.0	0.0	0.0	0.0	0.0	0.0
	*An. funestus* s.s	0.5	1.6	32.0	0.0	0.2	3.0	0.0	0.1	1.0
	*An. cf. coustani*	0.0	0.0	0.0	0.0	0.0	0.0	0.0	0.1	1.0
	Mean ± SD	5.0 ± 9.7	17.2 ± 26.8	355.0 ± 582.5	0.6 ± 1.2	3.2 ± 6.3	61.0 ± 120.0	2.5 ± 5.0	7.6 ± 12.1	153.0 ± 304.7
Zambezi	*An. arabiensis*	0.7	1.6	37.0	0.3	1.5	28.0	0.1	0.4	9.0
	*An. gambiae* s.s	0.0	0.0	0.0	0.0	0.0	0.0	0.0	0.0	0.0
	*An. funestus* s.s	0.0	0.0	0.0	0.0	0.0	0.0	0.0	0.0	0.0
	*An. cf. coustani*	0.00	0.0	0.0	0.0	0.0	0.0	0.0	0.0	0.0
	Mean ± SD	0.2 ± 0.3	0.4 ± 0.8	9.3 ± 18.5	0.1 ± 0.1	0.4 ± 0.8	7.0 ± 14.0	0.0 ± 0.1	0.1 ± 0.2	2.3 ± 4.5
Ohangena	*An. arabiensis*	0.3	0.1	7.0	0.1	0.2	4.0	0.0	0.1	1.0
	*An. gambiae* s.s	0.0	0.0	0.0	0.0	0.0	0.0	0.0	0.0	0.0
	*An. funestus* s.s	0.5	0.6	18.0	0.0	0.0	0.0	0.0	0.0	0.0
	*An. cf. coustani*	0.0	0.0	0.0	0.0	0.0	0.0	0.0	0.0	0.0
	Mean ± SD	0.2 ± 0.3	0.2 ± 0.3	6.3 ± 8.5	0.1 ± 0.0	0.1 ± 0.1	1.0 ± 2.0	0.0 ± 0.0	0.1 ± 0.0	0.3 ± 0.5
Kavango West	*An. arabiensis*	0.1	0.4	8.0	0.2	1.0	14.0	0.0	0.3	5.0
	*An. gambiae* s.s	0.0	0.0	0.0	0.0	0.0	0.0	0.0	0.0	0.0
	*An. funestus* s.s	0.0	0.0	0.0	0.1	0.1	2.0	0.0	0.1	1.0
	*An. cf. coustani*	0.0	0.0	0.0	0.0	0.0	0.0	0.0	0.0	0.0
	Mean ± SD	0.1 ± 0.1	0.1 ± 0.2	2.0 ± 4.0	0.1 ± 0.1	0.3 ± 0.5	4.0 ± 6.7	0.0 ± 0.0	0.1 ± 0.2	1.5 ± 2.4
Kunene	*An. arabiensis*	2.8	8.1	173.0	No samples collected					
	*An. gambiae* s.s	2.4	4.6	112.0						
	*An. funestus* s.s	0.1	0.0	1.0						
	*An. cf. coustani*	0.0	0.0	0.0						
	Mean ± SD	1.3 ± 1.5	3.2 ± 3.9	71.5 ± 85.7						
Omusati	*An. arabiensis*	2.1	10.9	208.0						
	*An. gambiae* s.s	0.0	0.0	0.0						
	*An. funestus* s.s	0.0	0.1	1.0						
	*An. cf. coustani*	0.0	0.0	0.0						
	Mean ± SD	0.5 ± 1.1	2.7 ± 5.4	52.3 ± 103.8						
Oshikoto	*An. arabiensis*	0.0	0.8	9.0						
	*An. gambiae* s.s	0.0	0.0	0.0						
	*An. funestus* s.s	0.0	0.0	0.0						
	*An. cf. coustani*	0.0	0.0	0.0						
	Mean ± SD	0.0 ± 0.0	0.2 ± 0.4	2.3 ± 4.5						
Otjizondjupa	*An. arabiensis*	0.0	0.0	0.0						
	*An. gambiae* s.s	5.5	11.7	275.0						
	*An. funestus* s.s	0.0	0.0	0.0						
	*An. cf. coustani*	0.0	0.0	0.0						
	Mean ± SD	1.4 ± 2.8	2.9 ± 5.8	68.8 ± 137.5						
National	*An. arabiensis*	25.8	78.1	1662.0	2.8	15.2	287.0	10.1	28.9	625.0
	*An. gambiae* s.s	8.0	26.6	553.0	0.0	0.0	0.0	0.0	0.0	0.0
	*An. funestus* s.s	1.0	2.3	52.0	0.1	0.3	5.0	0.0	0.1	2.0
	*An. cf. coustani*	0.0	0.0	0.0	0.0	0.0	0.0	0.0	0.1	1.0
	Mean ± SD	8.7 ± 11.9	26.7 ± 36.3	566.8 ± 771.6	0.7 ± 1.4	3.9 ± 7.6	73.0 ± 142.7	2.5 ± 5.1	7.3 ± 14.4	157.0 ± 312.0

*bpn* Bites per night, *HBR *human biting rate, *SD* standard deviation, * s.l.* sensu lato, * s.s.* sensu stricto

Overall, *An. arabiensis* was the primary vector (indicative of HBR) caught at most sites and sampling points (Table 1). The only site and time point where *An. arabiensis* were not sampled was Otjozondjupa in 2018. *Anopheles gambiae* s.s. was the second most common vector and was found in 2018 in Kavango East, Kunene and Otjozondjupa. *Anopheles funestus* s.s. was seen in Kavango East, Ohangwena, Kavango West, Kunene and Omusati in 2018. *Anopheles gambiae* s.s. was absent in collections from 2019, while *An. cf. coustani* was only sampled in 2019 in Kavango East.

### Biting time

**Fig. 2:**
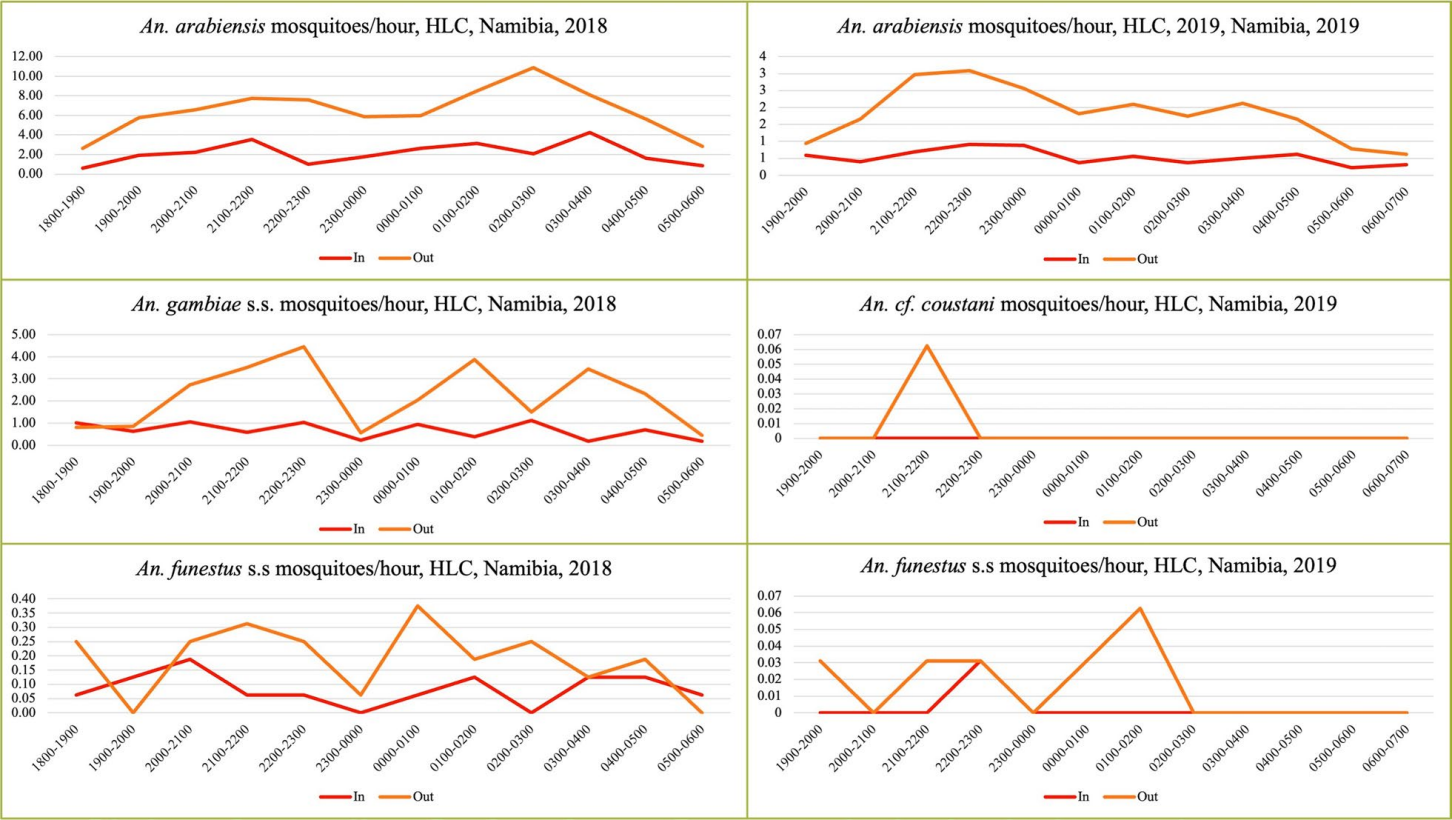
Vector species biting times across nights during the 2018–2019 entomological surveillances. HLC, Human landing catch; In, indoors; Out, outdoors; s.s., sensu stricto

*Anopheles* mosquitoes were found biting humans (in HLCs) from dusk until dawn both indoors and outdoors during both collection rounds and time points. Generally* An. arabiensis* had the highest peak biting times, between 0200 hours and 0300 hours outdoors and between 0300 hours and 0400 hours indoors in 2018. However, in 2019, peak biting times for the same vector species was observed before midnight, between 2200 hour and 2300 hours for both outdoor and indoor locations (Fig. 2).

### Biting location

As a population, all anophelines were generally more exophagic than endophagic (*β* = 1.04, *P* < 0.001), and this tendency increased in 2019 (*β* = 0.33, *P* = 0.02). Biting location explained a significant amount of variation among the total of each species caught (*P* < 0.05), but *An. gambiae* s.s. (*β* = 1.34) and An. arabiensis (*β* = 1.11) were more exophagic than *An. funestus* s.s*.* (*β* = 0.76).

Overall, in 2018, 75% (*n* = 1711) of the *Anopheles* mosquitoes were captured outdoors and 25% (*n* = 556) were captured indoors. Similarly, in 2019, the proportion of mosquitoes captured outdoors (77.5%, *n* = 713) was higher than those captured indoors (22.5%, *n* = 207) in both round 1 and round 2. The indoor and outdoor HBRs are presented for the three primary vectors, namely *An. arabiensis*, *An*. *gambiae* s.s. and *An. funestus s.s.*, with *An*. *cf. coustani* as a secondary vector, in Table 1.

### Indoor resting behavior

The PSCs were used to estimate indoor resting behavior (endophily) in 2019. All specimens sampled were identified as *An. arabiensis* using PCR. Indoor resting rates were less than outdoor biting rates. Overall, in Kavango East, only 0.1% of *An. arabiensis* that targeted humans (indoors and outdoors) (25 specimens found in PSCs vs 856 found in HLCs) were found resting indoors in the morning (Table 2). The indoor resting rate for Kavango West and Ohangwena was low (< 1 mosquito per structure); the highest indoor resting rate was in Zambezi region, with a low indoor resting rate of two mosquitoes per structure. By linking resting and biting behaviors, the proportion of mosquitoes that IRS may effectively target can be determined as follows:

**Table 2:** Indoor resting rates (mosquitoes per structure) and minimum estimated indoor residual spraying effectiveness

Region	Trap type	*Anopheles*/trap/night (2019)					Minimum estimated IRS effectiveness
		Round 1		Round 2		Total	
		Indoors	Outdoors	Indoors	Outdoors		
**Kavango East**	HLC	0.57	3.23	2.50	7.07	856	0.14 (mosquitoes/person)
	PSC	0.44	Na	4.5	Na	25	
**Kavango West**	HLC	0.06	0.27	0	0.09	22	NA
	PSC	0.25	Na	0	Na	1	
**Ohangwena**	HLC	0.02	0.05	0	0.02	5	
	PSC	0.50	Na	0	Na	2	
**Zambezi**	HLC	0.06	0.38	0.03	0.11	37	
	PSC	2	Na	1	Na	12	

*HLC* Human landing catch,* IRS* indoor residual spraying,* PSC* pyrethrum spray catch, *NA* not applicable

$$Minimum\,estimated\,IRS\,effectiveness\, = \,Mean\,\# \,of\,mosquitoes\,resting\,indoors/\left[ {Mean\,\# \,of\,mosquitoes\,biting\,indoors\, + \,Mean\,\# \,of\,mosquitoes\,biting\,outdoors} \right],$$

expressed as the number of mosquitoes/person. This indicator describes the proportion of mosquitoes that are found resting on indoor surfaces (potentially sprayed and possibly killed by IRS) out of all mosquitoes that were observed to bite in the community (both indoors and outdoors, as a measure of vector density). Values > 1 indicate that more mosquitoes are found resting than biting; values < 1 indicate that more mosquitoes are found biting than resting. The minimum estimated IRS effectiveness determined for Kavango East was 0.14 mosquitoes/person, with too few samples from the other sites for the calculation (Table 2).

Indoor resting rates (mosquitoes per structure) and minimum estimated IRS effectiveness were determined in 2019. All specimens were determined to be *An. arabiensis*.

RBCs (data not shown) were also conducted both indoors and outdoors in 2018 towards evaluating sampling methods. An insignificant number (*n* = 11) of *Anopheles* were found in RBs both indoors or outdoors across the 248 sampling nights conducted.

### Insecticide resistance

**Fig. 3:**
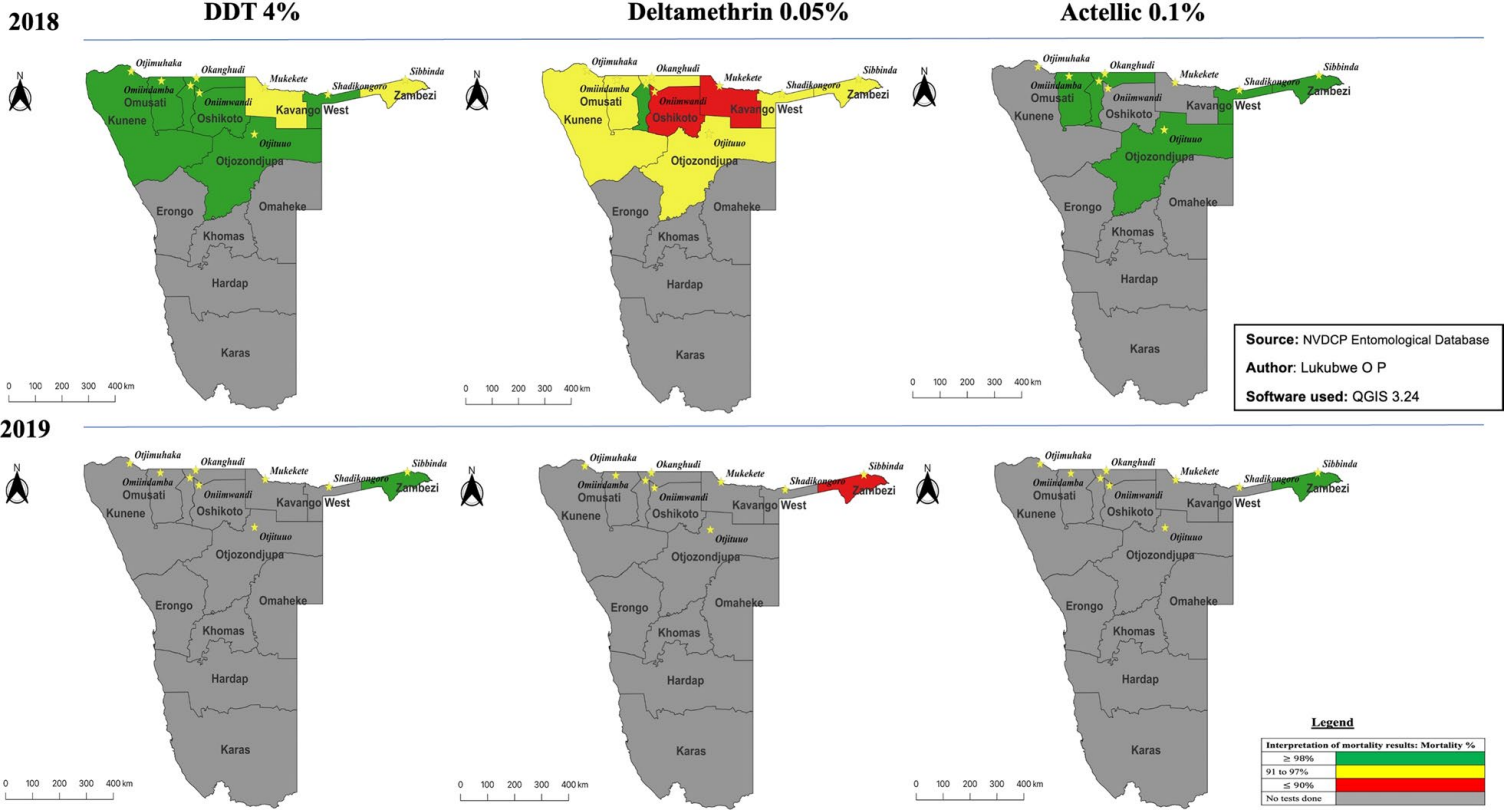
Insecticide susceptibility status, Namibia 2018–2019. DDT, Dichlorodiphenyltrichloroethane; NVDCP, National Vector-borne Diseases Control Program

In 2018 the overall mortality of *An. gambiae* s.l. mosquitoes following exposure to diagnostic doses of deltamethrin ranged from 91.3% to 95.6% for Kunene Ohangwena, Omusati, Otjozondjupa, Kavango East and Zambezi, indicating possible resistance in the populations according to WHO criteria. There was confirmed resistance in mosquitoes sampled from Oshikoto and Kavango West, with those from Oshana being susceptible (Fig. 2). The *An. gambiae* s.l. mosquitoes from all sentinel sites were susceptible to DDT, with the exception of those from Kavango West and Zambezi that demonstrated possible resistance (Fig. 3). *Anopheles gambiae* s.l. mosquitoes from all sites were susceptible to pirimiphos-methyl (Actellic 300CS).

A subset of *An. gambiae* s.l. mosquitoes (*n* = 1524) were identified by molecular methods to species level; of these, 61.1%, (*n* = 931) were *An. gambiae* s.s. and 39.9% (*n* = 593) were *An. arabiensis*. *Anopheles gambiae* s.s. (79.1%, *n* = 34) was more abundant among the samples that showed possible signs of resistance (alive), with *An. arabiensis* accounting for 29.9% (*n* = 9). (Fig. 3).

**Fig. 4:**
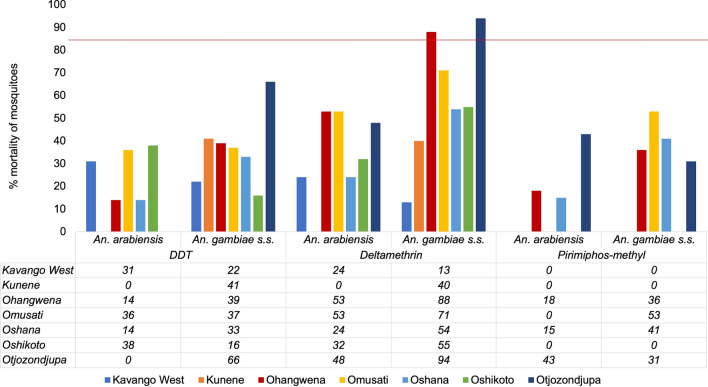
Insecticide resistance of *Anopheles* mosquitoes (% mortality) according to species composition, by site and insecticide, Namibia 2018. DDT, Dichlorodiphenyltrichloroethane, s.s., sensu stricto

During the 2019 entomological surveillance, IR tests were conducted for only one sentinel site (Zambezi) due to insufficient larvae sampled. Susceptibility status at this site changed from possible resistance (95.6% in 2018) to confirmed resistance (80.0%) for deltamethrin while the susceptibility status of DDT and Actellic remained at 100.0% susceptible (Fig. 4).

A subset of *An. gambiae* s.l. mosquito samples (*n* = 320) from 2019 were sent for PCR analysis. Among these samples, *An. arabiensis* was the most abundant, accounting for 93.4% (*n* = 299), followed by *Anopheles quadriannualatus* (3.8%, *n* = 12) and *Anopheles* ‘other’ (unknown) (2.8%, *n* = 9). In addition, all of the samples that showed possible resistance (alive) were identified as *An. arabiensis* (75.0%, *n* = 15), *An. quadriannualatus* (5.0%, *n* = 1), *Anopheles* ‘other’ (20.0%, *n* = 4), of which were all exposed to deltamethrin.

## Discussion

Namibia has scaled up vector control interventions over the last two decades,which has had a demonstrated impact on disease [6]. Despite routine assessments on the impact of IRS on epidemiological outcomes [36, 37], the impact of the IRS strategy on vector species and bionomic traits, which is the target of IRS, has never been investigated. The ESPT [38] was used to select minimum essential indicators, design a sampling strategy and analyze and interpret data. Evidence gathered was directed at understanding key minimal essential indicators that describe and point to drivers of transmission. These indicators were also directed at understanding appropriate sampling methods and the efficacy of ongoing IRS programs. Overall, the evidence generated may be used in programmatic decision-making on intervention strategy and resource allocation by understanding gaps in protection.

Entomological surveillance in the 2018 study revealed the presence of *An. arabiensis*, *An. gambiae* s.s., *An. funestus* s.s. and secondary *Anopheles* species. The re-emergence of the primary anthropophagic and endophagic vector *An. gambiae* s.s. found in previous studies [11] is of considerable importance since it was thought to have disappeared in the mid-2000s. Both the disappearance and the re-appearance of this species may be associated with insecticide use. The initial disappearance may have resulted from an intervention-mediated impact on the susceptible species, resulting in a population decline similar to that seen in Kenya [39], while the application of the same IRS insecticide over several years may have selected for or allowed the re-invasion of insecticide-resistant *An. gambiae* s.s. [40], the presence of which was documented in this study. In contrast to 2018, *An. gambiae* s.s. and *An. funestus* s.s. were not found the following year (2019), possibly due to the severe drought experienced in 2019 as the populations of these species are known to decline significantly during dry periods [41].

*Anopheles arabiensis* is predominantly found in arid areas with low rainfall, is both endophagic and exophagic and is widely distributed in the northern regions of the country [1, 6]. This results in multiple gaps in protection based on the interventions currently present and insecticide resistance, along with human behaviors, making this species less responsive to control by conventional vector control tools [3, 6, 11]. These factors explain both its continued presence in Namibia and its contributions to disease transmission.

Interestingly both the 2018 and 2019 entomological monitoring identified other *Anopheles* species (i.e. *An. coustani* s.l. and *An. squamosus*). Although these vectors are largely zoophilic and exophagic, recent studies have found high anthropophagic behaviors in these vectors, with blood meals from humans, and the presence of *Plasmodium* parasites [42]. Their potential role in disease transmission needs to be further investigated. Based on its presence, density and biting behavior, *An. arabiensis* may be the primary vector that contributes the most to transmission. The presence of three primary vectors (*An. arabiensis*, *An. gambaie* s.s. and *An. funestus* s.s.) along with secondary vectors, all with variable behavior and temporal presence, suggests a transmission system that adapts to a dynamic environment. Taken together, this points to the need for continued routine entomological surveillance directed at a strategic plan that adjusts to changing transmission dynamics.

*Anopheles arabiensis* is known to be exophilic and exophagic [3, 6, 11], as demonstrated by the data in this study. Its host-seeking behavior throughout the night, both indoors and outdoors, demonstrates that interventions need to be effective throughout the night when this species is the target. Despite *An. gambiae* s.s. and *An. funestus* s.s. being conventionally known to be endophagic [43–45], our results demonstrated that there was more outdoor biting than indoor biting by these species. This shift from primarily indoor biting to outdoor biting demonstrates the adaptive nature of this species and may also be associated with the increasing selective pressure of indoor insecticides. This change also points to the importance of temporal entomological data and the need for adaptive intervention strategies.

Overall, *Anopheles* host-seeking was documented throughout the night in both 2018 and 2019, indicating the need for all-night protection. When the presence of multiple vectors was considered, outdoor biting was higher than indoor biting. Outdoor biting mosquito populations contribute to malaria transmission in many parts of sub-Saharan Africa and pose new challenges as they cannot be reliably monitored or controlled using conventional tools [46]. The overall biting activities across the night of the major vector species had two distinctive peaks, one before midnight and another after midnight. Changes in vector behavior associated with indoor interventions have been demonstrated and now need to be expected in multiple other sites [47, 48]. However, these outdoor biting behaviours do not reduce the importance of indoor interventions as primary exposure to and impact of mosquito populations may still occur indoors [49, 50].

Overall, outdoor biting was higher than indoor biting in both 2018 and 2019. This increased outdoor biting behavior of vectors may adversely affect the effectiveness of conventional vector control interventions like LLINs [46, 51].

The efficacy of IRS is dictated by the indoor resting rate of the targeted species. Our data demonstrated that indoor resting was limited to a small proportion of the overall *An. Arabiensis *population, a possible outcome of previously successful IRS implementation that selected for resistant behaviors. The minimum estimated IRS effectiveness of 0.14 indicates that IRS would impact only 14% of mosquitoes that bite humans and then rest on walls. This estimation does not factor in IR, which may further lower this efficacy. Hourly indoor aspirations combined with window exit traps would determine if PCSs missed vectors that may enter and leave before dawn, data that would enable the determination of further IRS protections. It should be noted that outdoor resting boxes were inefficient at capturing or determining outdoor resting densities.

IR in 2018 to the primary insecticide (deltamethrin) used in the interventions was confirmed at some sites and demonstrated to be possible or to be developing at other sites. Data from 2019 demonstrated an increase in resistance to deltamethrin at the single site where testing was conducted (Zambezi). This increasing resistance is a warning of possible further reductions in intervention efficacy and validates the need for a further scale-up and routine IR tests. The demonstration of susceptibility to other insecticides can also steer the program towards a shift in insecticides as a response to the observed seen. These insecticides may be used in a rotation or mosaic system to combat the further development of resistance while maintaining intervention efficacy [16].

An interesting observation and outcome of this study is that CDC-LTs were not an effective sampling tool at these sites, with mosquito catches not being representative of human exposure, as demonstrated by HLCs. In addition, CDC-LTs did not capture the most abundant species—*An. arabiensis*—either indoors or outdoors in Kavango East, pointing to a further limitation when using this tool at these sites. This data demonstrate that the selection of a sampling tool implicitly impacts both the data and downstream analysis, illustrating the necessity of validating sampling tools within the local context based on the question before scale-up for national use towards decision-making.

This 2-year operational surveillance observational report has multiple drawbacks, demonstrating the standard limitations of national implementation programs based on both funding and capacity. Further support would greatly enhance both the significance and the applicability of this data in decision-making. Primary limitations include the sampling frame where additional sampling (i.e. more structures, more collection nights, etc.) would enhance the representativeness of the data. Limited funding also restricted the number of samples that could be identified to species level using molecular methods.

Despite these limitations, the ESPT-based framework utilized a question-based approach towards selecting indicators, evaluating tools that were used to collect indicators, alongside using the program capacity that both determined and directed the surveillance strategy, thereby answering relevant program questions. Direct responses to the data from this ESPT-formulated entomological surveillance include the elimination of CDC-LTs from local sampling, the operational evaluation of alternative insecticides for IRS, the evaluation of the impact of IRS duration on various wall types and routine yearly entomological surveillance strategies that build on previous data.

## Conclusions

Key outcomes of this study include the identification of the primary vector *An. gambiae* s.s. and species-specific bionomic traits that impact and characterize intervention effectiveness, such as spatial and temporal biting (impacting LLINs) and indoor resting (impacting IRS). IR assays also documented the increase of resistance at sentinel sites. Baseline evaluation of sampling tools demonstrated that the CDC-LTs and resting boxes were not optimal sampling methods for these sites. Targeted and question-based entomological surveillance enabled a rapid and focused evidence base on which the Namibian Ministry of Health and Social Services may further build a monitoring and evaluation framework towards understanding drivers of transmission.

## Data Availability

The data and materials that support the findings of this study are available from the corresponding author upon reasonable request.

## Abbreviations

*An. arabiensis*: *Anopheles arabiensis*
*An. coustani*: *Anopheles coustani*
*An. funestus s.l*: *Anopheles funestus sensu lato*
*An. funestus s.s*: *Anopheles funestus sensu stricto*
*An. gambiae s.l*: *Anopheles gambiae sensu lato*
*An. gambiae s.s*: *Anopheles gambiae sensu stricto*
*An. quadriannualtus*: *Anopheles quadriannualtus*
*An. squamosus*: *Anoppheles squamosus*
bpn: Bites per night
CDC-LTs: Centers for Disease Control and Prevention light traps
Co1: Cytochrom C oxidase subunit 1
CVs: Community Volunteers
DDT: Dichlorodiphenyltrichloroethane
E8: Elimination 8 (eight)
ESPT: Entomological Surveillance Planning Tool
GLMM: Generalized Linear Mixed Models
HBR: Human biting rate
HLCs: Human landing catches
IR: Insecticide resistance
IRS: Indoor residual spraying
ITS2: Internal Transcribed Spacer 2
LSM: Larval Source Management
LLINs: Long-lasting insecticidal nets
NA: Not Applicable
NVDCP: National Vector-borne Diseases Control Program
PCR: Polymerase Chain Reaction
PSCS: Pyrethrum spray catches
RBCs: Resting box collections
SD: Standard Deviation
WHO: World Health Organization
